# Single-cell profiling identifies heterogeneity of the immune microenvironment in healthy, primary and lymph node metastatic BC

**DOI:** 10.3389/fimmu.2025.1690992

**Published:** 2025-11-27

**Authors:** Chuyu Liu, Feng Zeng, Shanshan Gao, Nengying Zhang, Yulan Cai

**Affiliations:** 1 Thyroid and Breast Surgery, The Second Affiliated Hospital of ZunYi Medical University, Zunyi, Guizhou, China; 2 Department of Biomedical Sciences, Faculty of Health Sciences, University of Macau, Taipa, Macau SAR, China; 3 Department of Endocrinology and Metabolism, The Second Affiliated Hospital of Zunyi Medical University, Zunyi, Guizhou, China

**Keywords:** breast cancer, lymph-node metastasis, single-cell RNA sequencing, endothelial cell heterogeneity, immune microenvironment

## Abstract

**Introduction:**

Breast cancer (BC) prognosis is fundamentally dictated by its pronounced molecular and cellular heterogeneity. While single-cell RNA sequencing (scRNA-seq) has detailed the tumor microenvironment (TME) cellular composition, a critical knowledge gap persists regarding the vascular heterogeneity of tumor endothelial cells (TECs) across different BC subtypes. Current TEC classification remains conventional, obscuring whether subtype-specific TECs possess unique transcriptomic signatures that influence treatment response, particularly to anti-angiogenic therapies. Given the pivotal role of tumor vasculature in proliferation and metastasis, and the existing research bias towards Triple-Negative BC (TNBC), a comprehensive investigation into the complexity of the vascular system is warranted. Furthermore, as lymph node metastasis (LNMT) is a major determinant of cancer mortality, distinguishing the endothelial and immune cell subtypes within LNMT microenvironments from primary tumors is essential for clarifying pathogenesis.

**Methods:**

To address this, we integrated scRNA-seq data from primary BC tumors, ER_LN and healthy tissues, generating a robust dataset of 98,000 cells (~ 12 samples). Employing single-cell transcriptomics, spatial transcriptomics, and immunohistochemistry, we precisely delineated the endothelial cell (EC) heterogeneity within matched tumor and peri-tumoral tissues and scrutinized their intricate interactions with immune populations.

**Results:**

We identified two previously uncharacterized, tumor-enriched endothelial cell subtypes, designated EC4 and EC5, which demonstrate subtype-specific functional adaptations and prognostic significance. EC4 cells, highly prevalent across BC, are principally characterized by antigen presentation, immune cell recruitment, and pro-inflammatory signaling. Conversely, EC5 cells, also enriched in BC, exhibit robust extracellular matrix (ECM) remodeling and potent tumor angiogenesis. We further characterized the functional divergence of EC4 and EC5 within ER tumors relative to HER2 tumors and ER_LN metastases. Our analysis reveals conserved endothelial programming mechanisms across BC subtypes, coexisting with distinct TME-driven transcriptional adaptations. Importantly, interactome analysis highlighted novel and subtype-specific communications between these novel EC subsets and immune cells, particularly CD8+T cells and macrophages. Experimental validation demonstrates that ECs overexpressing APP can mediate the M2 polarization of macrophages, underscoring diverse immunomodulatory roles for EC subsets across different BC contexts.

**Conclusions:**

These findings offer critical and granular insights into the complex interplay between novel EC subtypes and the immune microenvironment in BC progression and metastasis. We establish that ECs are active and heterogeneous modulators of the TME, identifying specific therapeutic vulnerabilities within the tumor vasculature and providing a foundational blueprint for developing future precision immunotherapeutic strategies.

## Introduction

Breast cancer (BC) represents a major global health challenge, affecting millions of women worldwide. While established histological and phenotypic markers are crucial for prognostic assessment and treatment stratification, the clinical impact of BC’s intra-tumoral heterogeneity is increasingly recognized as a determinant of patient-specific outcomes. The molecular features of BC display pronounced heterogeneity across its various subtypes, thereby mandating customized therapeutic regimens (1). Thus, delineating the composition of distinct BC types bears significant clinical import. Single-cell RNA sequencing (scRNA-seq) has profoundly enhanced our understanding of both inter- and intra-tumoral cellular diversity within BC, successfully delineating various tumor cell states and characterizing key stromal components, including immune cells, myoepithelial cells, and fibroblasts. However, a comprehensive appreciation of vascular heterogeneity in BC remains relatively limited, often constrained to conventional EC subtypes (e.g., angiogenic, arterial, capillary, venous, and lymphatic). It remains unclear whether tumor EC (TECs) from distinct tumor types exhibit diverse phenotypes and possess unique transcriptomic signatures, which may differentially influence their response to anti-angiogenic therapies. At present, there is a deficiency in systematic research on the interactions among various cell types within the tumor microenvironment. Existing studies in this field have primarily emphasized triple-negative BC (TNBC) (2–4). Given that tumor blood vessels are critical conduits for tumor proliferation, local invasion, and distant metastasis, a deeper elucidation of the heterogeneity within the vascular system of different BC subtypes and its complex interplay within the tumor microenvironment TME represents a critical and currently unmet need in the fields of cancer biology and therapeutic development. Metastasis stands as the predominant cause of cancer-associated morbidity and mortality. Owing to the unique architecture of lymph node (LN) vessels in tumors, tumor cells exhibit a propensity to metastasize to LN tissue, with LN metastasis serving as an early indicator of metastatic tumors (5). The microenvironment of lymph node metastasized tumors (LNMTs) is posited to be immunosuppressive. However, the defining features and specific mechanisms of the diverse immune cells within this microenvironment remain elusive. Research has demonstrated that metastatic LNs can modulate tumor immune responses. For instance, targeting metastatic LNs can markedly enhance the therapeutic efficacy for primary tumors, suggesting that this approach may be a crucial strategy for improving patient survival rates (6). Consequently, it is of paramount importance to delineate and distinguish between the microenvironments of LNMTs and primary tumors.

In this study, we employ single-cell transcriptomic datasets to enhance our comprehension of how EC might impact BC. We concentrate on ER,HER2, and ER_LN, delving into the heterogeneity of EC within matched tumoral and peri-tumoral tissues with greater precision. Moreover, we scrutinize the interactions between EC and immune cell populations. These cellular interactions hold potential therapeutic significance, particularly in the context of immunotherapy, as EC serve as a pivotal interface with the immune system, yet they have been underexplored in prior BC scRNA-seq investigations (7, 8). Our findings also expose two EC subtypes that are prevalent in tumoral breast tissues and may facilitate malignant cell metastasis and affect patient prognosis, with an emphasis on unraveling their distinctions. Through our analyses, we demonstrate that the immunomodulatory capacity and tumor micro environment-shaping potential of EC differ among various BC subtypes (6, 9–12). Our data offer novel perspectives on tumor development mechanisms and immune infiltration by focusing on the less-studied EC, which could be instrumental for the clinical application of immunotherapy targeting BC progression and metastasis.

## Result

### Cellular composition of primary and metastatic BC

**Figure 1:**
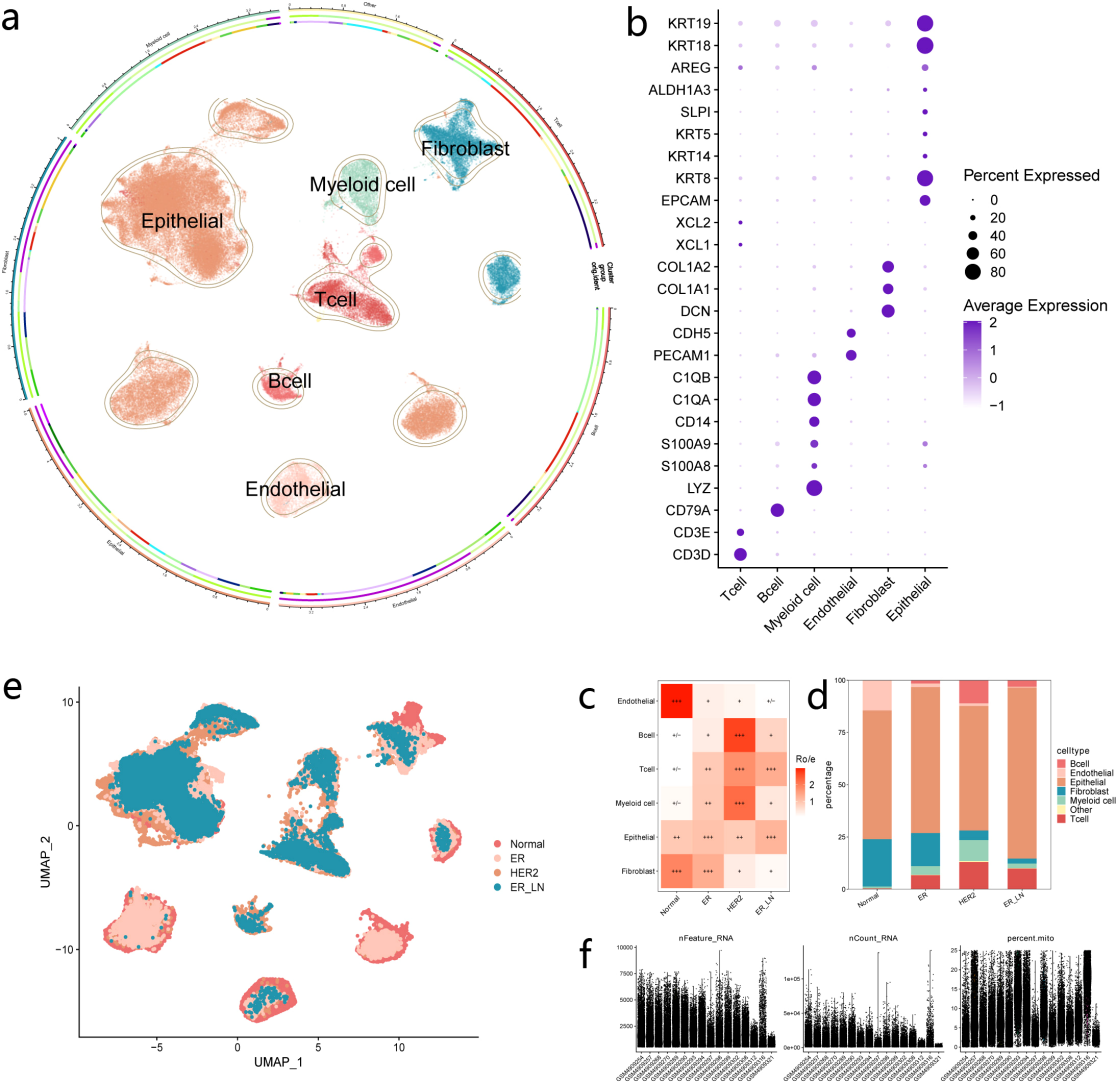
The major cell types revealed by the different BC subtypes. **(a, e)** UMAP embedding plot showing identified clusters of all cells from Healthy,ER,HER2 and lymph-node metastases of ER of 16 patients. Cells were colored according to their cell type **(a)** or cancer subtypes **(e)**. **(b)** Dot plot showing the marker gene expression for each major cell type in **(a)**. **(c)** Heatmap showing the BC subtypes distribution of each major cell type by Ro/e analysis. BC-enriched types were characterized with Ro/e > 1.[Ro/e > 1]: +++;[0.8 < Ro/e <= 1]: ++;[0.2 <= Ro/e <= 0.8]: +;[0 < Ro/e < 0.2]: +/-;[Ro/e = 0]:-.Fisher’s exact test was used to compare significance. **(d)** The major cell type proportions among the BC subtypes. Colors on the columns indicate cell type. **(f)** Violin plots showing the number of counts, the number of genes and the ratio of mitochondrial genes in each cell of each sample after quality control.

In this study, we aimed to uncover the cellular and molecular characteristics of different BC subtypes. To this end, we collected samples from four healthy individuals without BC (4 Normal),eight primary tumors (4 ER and 4 HER2),and four ER_LN (4 ER_LN) from 12 BC patients for scRNA-seq analyses. The primary BC cohort comprised four ER and four HER2 BC samples. A total of 98000 single-cell transcriptomes were acquired. Following rigorous quality-control procedures,23971 cells from ER,27143 cells from HER2,19848 cells from ER_LN, and 27038 cells from normal individuals were retained for further analysis (**Figure 1f**, **Supplementary Table 1**).

Unsupervised graph-based clustering integrated all the cells and identified six major cell types (**Figures 1a, e**), which were annotated based on established cell-type-specific markers (**Figure 1b**). In terms of immune cells, T cells were identified by the expression of CD3D,CD3E; myeloid cells were marked by CD14and LYZ; and B cells were recognized by CD79A.As for non-immune cells, EC were labeled by CDH5 and PECAM1; fibroblasts were identified by DCN,COL1A1 and COL1A2; and epithelial cells were marked by EPCAM and KRT18 (**Figure 1b**).

To further explore the composition of different cell types in ER,HER2 and ER_LN, we assessed these tumors against normal tissues from healthy patients. Using Ro/e analysis (13) to compute the enrichment scores, which measured the proportion of each cell type in normal patients compared to primary tumors or lymph-node metastatic tumors, we sought to quantify the differences in cell type distribution (**Figure 1c**).The analysis revealed that B cells, T cells and myeloid cells were significantly enriched in the tumor microenvironment, reflecting the infiltration of lymphoid and myeloid cells. Notably, in BC tumor patients, EC were less abundant compared to normal patients (**Figures 1c, d**). This observation suggests that EC might play a crucial part in tumor formation and immune microenvironment remodeling.

### Characterization of EC in the BC microenvironment

**Figure 2:**
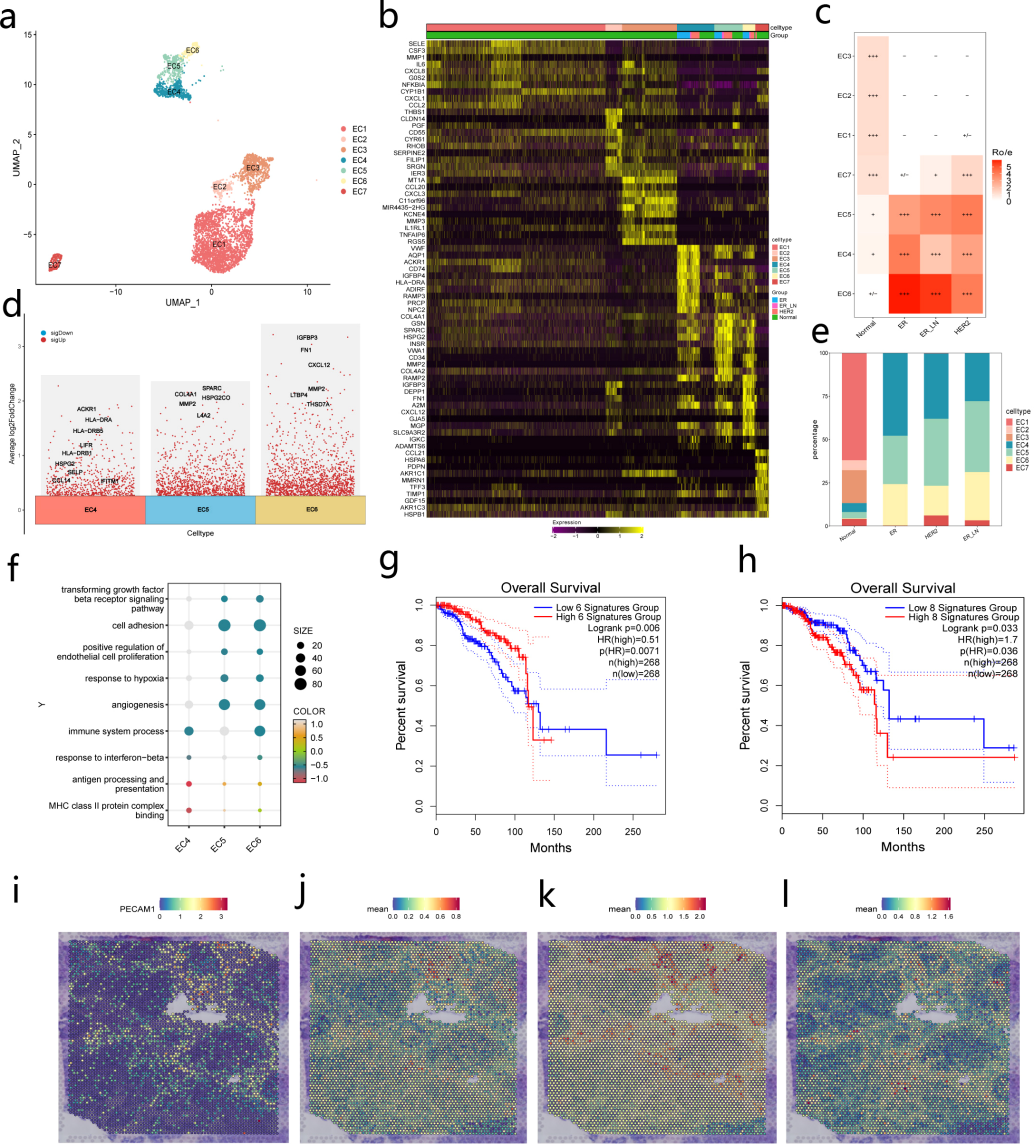
Single cell taxonomy of EC in the BC. **(a)** UMAP-plot of the subclustering of EC from Normal,ER,HER2 and ER_LN BC subtypes. **(b)** Heatmap of the expression levels of the top-10 marker genes in all 7 endothelial subclusters. Color scale: yellow – high expression, purple– low expression. EC:endothelial cell. **(c)** Heatmap showing the BC subtypes distribution of each major endothelial cell type by Ro/e analysis.[Ro/e > 1]:+++,[0.8 < Ro/e <= 1]:++,[0.2 <= Ro/e <= 0.8]:+,[0 < Ro/e < 0.2]: +/-,[Ro/e = 0]:-. **(d)** Scatter plot showing scaled expression of discriminative gene sets for EC4 EC5 and EC6 subsets. **(e)** Bar chart showing the relative proportion of each EC subset from different BC subtypes. Colors on the columns indicate cell type marked by legend. **(f)** Representative GO terms pathway enriched by upregulated differentially expressed genes of each EC subset. **(g)** Kaplan-Meier plots showing the survival probability of BC patients with high and low expression levels of EC4 signature genes. HR,hazard ratio.Red line indicates patients expressed higher EC4 signature genes, blue line indicates patients expressed lower EC4 signature genes. P value was calculated by log-rank test.HR,hazard ratio. **(h)** Kaplan-Meier plots showing the survival probability of BC patients with high and low expression levels of EC5 signature genes. (**i–l)**: Visualization of the expression of endothelial cell marker genes and endopia-characteristic genes in ST-seq.

There is accumulating support for the notion that EC could play a part in the altered immune stability within the context of malignancy (14–16). In our dataset, seven distinct types of EC were identified, classification relied on the top 50 marker genes, marker genes identifying individual clusters, and previously defined gene signatures sourced from our own generated EC taxonomies (17, 18), designated as EC1-EC7 (**Figure 2a**, **Supplementary Figure 2a**). These included three subtypes of normal breast EC (nECs): EC1 cells, characterized by SELE+ and CSF3+ expression; EC2 cells, marked by THBS1+ and PTGS2+ expression; and EC3 cells, defined by MMP3+ and CCL20+ (**Figure 2b**, **Supplementary Table 2**). Additionally, three subtypes of tumor-associated EC (tECs) were enriched in BC patients: EC4 cells, identified by ACKR1+ (19–21) and HLA-DRA+ expression (14, 22); EC5 cells, characterized by COL4A1+ and INSR+ expression; and EC6 cells, defined by IGFBP3+ and CXCL12+ expression. Furthermore, one subtype of lymphatic endothelium was identified: EC7 cells, marked by CCL21+ and PROX1+ expression (**Figure 2b**, **Supplementary Table 2**). The enriched genes for each endothelial cell subtype are shown in **Figure 2b**.

Compared to other EC subtypes,EC4 exhibited elevated expression levels of multiple genes implicated in antigen presentation (HLADRB1,HLA-DRB5,and HLA-DRA),immune cell recruitment (SELP,LIFR, and ACKR1),and anti-tumor inflammation (CCL14,IFITM1) (**Figure 2d**). Notably, existing reports have indicated that ACKR1 is associated with prognosis (23). Consequently,we hypothesize that this subtype may have an impact on prognostic outcomes. It should be emphasized, however, that all discovered potential biomarkers necessitate confirmation through studies involving larger, distinct patient populations to determine their predictive capacity and practical application in treatment selection (24). To test this hypothesis, we evaluated whether this gene panel could effectively predict clinical outcomes. The results demonstrated that the characteristic genes of EC4 could be employed to predict poor prognosis in TCGA-BC datasets (**Figure 2g**). EC5 displayed high expression levels of genes related to ECM remodeling (COL4A1,COL4A2,HSPG2,MMP2,SPARC),which have been previously implicated in tumor angiogenesis. The characteristic genes of EC5 were associated with poor prognosis (**Figure 2h**). EC6 exhibited elevated expression of genes associated with angiogenesis and development (CXCL12,THSD7A,LTBP4),as well as genes related to cell growth and proliferation (IGFBP3,FN1,MMP2).Although the characteristic genes of EC6 did not exhibit significant prognostic value, they revealed a trend toward poor prognosis (**Figure 2d**, **Supplementary Figure 2b**).

Subsequent Gene Ontology (GO) analysis also unveiled enriched pathways associated with antigen processing and presentation, response to interferon-beta, and immune system processes in EC4, suggesting that EC4 may represent an endothelial cell phenotype actively engaged in responding to inflammatory cell infiltration into the tumor.EC5 demonstrated highly expressed pathways related to angiogenesis, response to hypoxia, and positive regulation of endothelial cell proliferation. Meanwhile, cell adhesion and transforming growth factor-beta receptor signaling pathways were enriched in EC6 (**Figure 2f**, **Supplementary Tables 3–5**).These distinct gene signatures of the various EC subtypes underscore their diverse functions within the tumor microenvironment.

In our investigation of EC subtypes within the context of BC pathogenesis, we observed a notable shift in EC composition between tumoral and normal tissues. Specifically, the proportions of EC1,EC2,and EC3 subtypes were diminished in tumor samples, whereas EC4,EC5,and EC6 subtypes were significantly enriched (**Figure 2c**). This enrichment was quantitatively assessed using Ro/e analysis, which revealed a higher ratio of these subtypes in tumor tissues compared to normal counterparts (**Figure 2e**). The enrichment of the three subpopulations of endothelial cells,EC4,EC5 and EC6 suggests that they may play a crucial role in the remodeling and progression of the tumor microenvironment. Taking into account the significant prognostic value of EC4 and EC5,the following section will focus on conducting functional analyses of these two cell types.

To elucidate the spatial distribution and potential prognostic implications of EC4 and EC5,we conducted spatial transcriptomic analyses focusing on their characteristic gene expressions. The results demonstrated that genes specific to EC4 (**Figure 2k**) and EC5 (**Figure 2l**) were predominantly expressed in EC proximal to tumor regions, aligning with known endothelial markers (**Figure 2i**). Conversely,EC1-specific genes, typically associated with normal tissues, exhibited reduced expression in these areas (**Figure 2j**). Further Immunohistochemistry (IHC) staining using BC tissue microarrays, we confirmed the expansion of EC4 and EC5 in the EC subtypes of BC (**Supplementary Figures 1a, b**). These findings suggest a strong association between EC4 and EC5 subtypes and BC, indicating their potential role as prognostic indicators.

**Figure 3:**
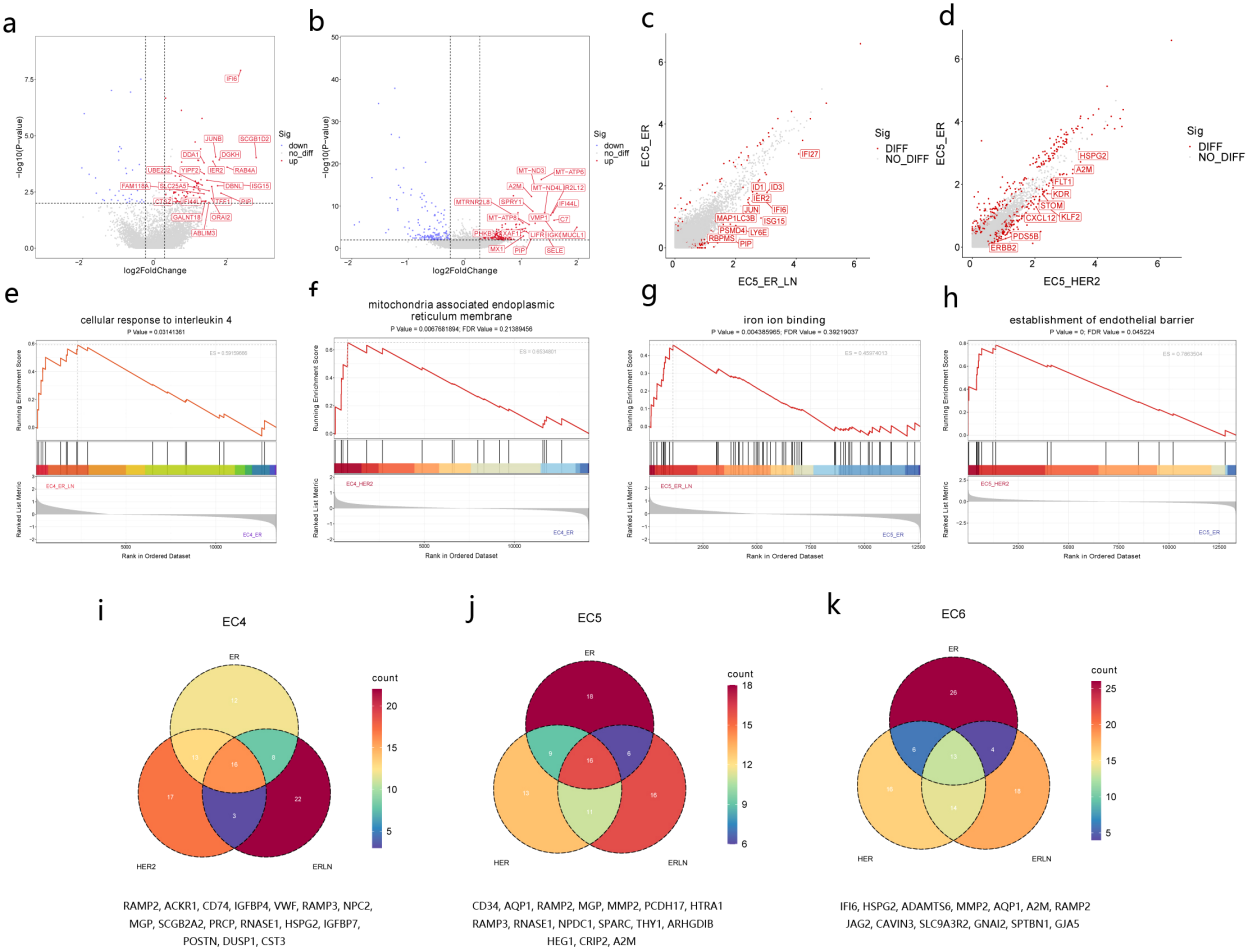
Characterization of EC subsets in BC subtypes. **(a)** Volcano plots showing upregulated DEGs of EC4 in ER_LN/ER comparison. **(b)** Volcano plots showing upregulated DEGs of EC4 in HER2/ER comparison. **(c)** Scatter plots showing upregulated DEGs of EC5 in ER_LN/ER comparison. **(d)** Scatter plots showing upregulated DEGs of EC5 in HER2/ER comparison. (**e–h)** GSEA analysis showing the enrichment pathway of DEGs in **(a–d)**.The colors of the line indicate the pathway of enrichment. (**i–k)** Venn diagram showing overlapping and unique genes of BC subtypes from EC4,EC5 and EC6.Overlapping genes are located at the bottom of the Venn diagram.

Further analysis into the heterogeneity of these prognostically relevant endothelial subpopulations across different BC subtypes was performed through differential gene expression (DEG) analyses. In ER_LN,EC4 cells exhibited upregulation of genes linked to immune response and inflammation (e.g.,SCGB1D2,IFI44L,IFI6),cell proliferation and differentiation (e.g.,JUNB,TFF1,CTSZ),as well as extracellular matrix remodeling and tissue repair (e.g.,GALNT18,EPAS1) (25) (**Figures 3a, e**, **Supplementary Figure 2c**, **Supplementary Table 6**). In HER2 tumors,EC4 cells showed increased expression of genes associated with antigen presentation and cell adhesion (e.g.,HLA-DRB1,SELL),along with genes involved in mitochondrial metabolism reprogramming (e.g.,MT-CO3,MT-ND4L,MT-ATP6) (26) (**Figures 3b, f**, **Supplementary Figure 2c**, **Supplementary Table 7**). These subtype-specific gene expression patterns underscore the functional diversity of EC4 and EC5 subtypes within the tumor microenvironment, highlighting their potential impact on tumor progression and patient prognosis.

In ER_LN,EC5 demonstrated significant upregulation of genes associated with type I interferon responses, including ISG15,IFI6,and LY6E (**Figures 3c, g**, **Supplementary Figure 2c**, **Supplementary Table 8**). This expression profile suggests an active role in immune modulation and potential mechanisms of immune evasion during lymphatic dissemination. Concurrently, autophagy-related genes such as MAP1LC3B and SQSTM1 were elevated, indicating enhanced autophagic activity that may facilitate cellular adaptation to microenvironmental stressors and contribute to tumor progression. Furthermore, the increased expression of RGS5,a regulator of vascular maturation, and FTH1,involved in iron metabolism, implies a supportive role for EC5 cells in maintaining endothelial integrity and remodeling the tumor microenvironment to favor metastatic colonization (27, 28).

In HER2,EC5 cells exhibited marked upregulation of angiogenesis-related genes, including KDR (VEGFR2),FLT1 (VEGFR1),and CXCL12,alongside components of the HER2 signaling pathway such as ERBB2 (29, 30). This gene expression pattern aligns with the aggressive vascular characteristics and therapeutic resistance commonly observed in HER2-enriched tumors. Additionally, elevated levels of HSPG2 (perlecan) and A2M (alpha-2-Macroglobulin) suggest enhanced interactions with the extracellular matrix, potentially compromising endothelial barrier function and posing challenges to effective drug delivery (31). Notably,both ER_LN and HER2 EC5 cells shared upregulation of RAP1B,a small GTPase, and SLC9A3R2,a scaffold protein, indicating common mechanisms that may facilitate endothelial migration and signal transduction across these subtypes (**Figures 3d, h** and **Supplementary Figure 2c**, **Supplementary Table 9**) (32, 33). In contrast,EC5 cells within ER lacking lymph node involvement did not exhibit these adaptive gene expression signatures, highlighting the subtype-specific nature of endothelial reprogramming. The consistent enrichment of ISG15 across different tumor subtypes underscores the activation of interferon-mediated pathways as a conserved feature of EC5,suggesting its potential utility as a biomarker for endothelial-driven immune modulation in BC (23).

To investigate the molecular signatures underlying disease progression across BC subtypes, we conducted a comparative intersection analysis of differentially expressed genes (DEGs) (34) identified in three endothelial cell subclasses (EC4, EC5 and EC6) derived from ER, HER2, and ER_LN breast malignancies relative to normal counterparts. The Venn diagrammatic representation demonstrated conserved transcriptional patterns across tumor subtypes, with 16 overlapping genes observed in EC4 populations,16 in EC5, and 13 in EC6 across all three cancer subtypes (**Figures 3i–k**). These conserved molecular signatures underscore the existence of subtype-independent endothelial programming mechanisms while simultaneously revealing distinct tumor microenvironment-driven transcriptional adaptations in breast carcinogenesis.

Our central hypothesis posits that endothelial marker genes exhibiting congruent expression patterns across multiple cancer types may represent superior candidates for pan-tumor anti-angiogenic therapeutic (AAT) targeting compared to tumor-specific vascular markers. Substantiating this premise (14), EC4 subclass analysis identified conserved functional modules including ECM remodeling effectors (HSPG2,MGP,POSTN) and tumor niche-modifying factors (RAMP2,ACKR1,CD74) (**Figure 3i**). In contrast,EC5 and EC6 subclasses exhibited conserved pro-angiogenic signatures encompassing established vascular morphogenesis regulators (CD34,AQP1) and emerging angiocrine signaling components (RAMP2/3,CRIP2,PCDH17) (**Figures 3j, k**),consistent with previous reports on tumor neovascularization dynamics (31, 35). His stratified molecular architecture not only validates known angiogenic pathways but also reveals subclass-specific therapeutic vulnerabilities across BC subtypes.

### Identification of myeloid cell subsets in primary and lymph-node metastases BC

**Figure 4:**
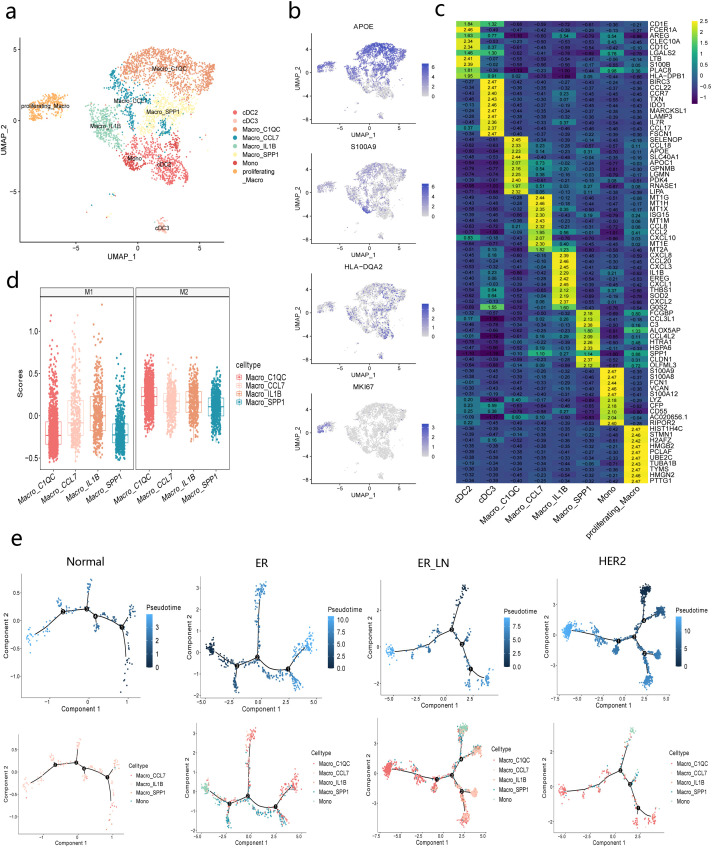
Myeloid cell functional analysis. **(a)** UMAP-plot of the subclustering of Myeloid cells from all BC subtypes. **(b)** UMAP plot displaying the marker genes of macrophages (APOE),DC (HLA-DQA2),Proliferating (MKI67)and monocytes (S100A9).Blue color indicates higher expression of these genes, pale grey indicates lower expression of these genes. **(c)** Heatmap displaying the expression of the top 10 differentially expressed genes of each Myeloid subclasses. Color scale: yellow-high expression, blue-low expression. **(d)** Module score analysis of M1/M2 macrophages between Macro_C1QC,Macro_CCL7,Macro_IL1B and Macro_SPP1 cell types. **(e)** Pseudotime analysis of selected Myeloid cell subsets for each BC subtypes. Top: distribution of the cells from different BC subtypes on each of the branches. Down: distribution of monocyte and macrophage subpopulations on each of the branches.

To delineate the functional heterogeneity of tumor-infiltrating myeloid cells-a pivotal immune compartment orchestrating critical oncogenic processes through divergent mechanisms such as therapy resistance and immune suppression (8)-we performed high-resolution transcriptional profiling across BC subtypes. Systematic clustering resolved eight transcriptionally distinct myeloid subsets (**Figures 4a, b**): five macrophage subclasses (Macro_C1QC, Macro_SPP1, Macro_IL1B,

Macro_CCL7,and Proliferating_ Macro),two dendritic cell populations cDC2 (defined by CD1C,IL1R2,and CLEC10A expression) and cDC3 (marked by CCR7 and LAMP3),and a monocytic lineage (FCN1,S100A8,S100A9).Subtype-defining biomarkers are comprehensively annotated in **Figure 4c**. Comparative analysis across tumor progression stages revealed dynamic compositional shifts between pro-inflammatory and resolution-phase macrophage subsets, suggesting functional polarization contingent on microenvironmental cues. Strikingly, we observed a pan-subtype enrichment of cDC2,Macro_C1QC,and Macro_SPP1 populations compared to normal tissue, forming a conserved immunoregulatory axis that may underlie metastatic niche formation and immune escape (**Supplementary Figures 2a, b**).

The cDC2 subset exhibited a paradoxical immunomodulatory phenotype, co-expressing antigen-presentation machinery (HLA-DPB1,HLA-DPA1,HLA-DQB1)and lipid-presenting CD1 family members (CD1C,CD1E) alongside immune-checkpoint regulators AREG (amphiregulin) and CXCL10.This duality implies concurrent activation of immunosuppressive pathways-potentially recruiting regulatory T cells (Tregs) via EGFR/STAT3 signaling-and suppression of cytotoxic T cell activity through CXCR3-mediated Th1/Th2 polarization defects.Notably,cDC2s uniquely expressed PLAC8,a metastasis-associated oncogene linked to PI3K/AKT pathway activation, suggesting their direct tumorigenic role beyond canonical immune regulation (36, 37) (**Figure 4c**, **Supplementary Table 10**).

Within the tumor microenvironment,Macro_C1QC undertakes a wide array of roles. It secretes chemokines such as CCL18 and complement components like C1QA,C1QB,and C1QC,thereby modulating immune responses and inflammatory processes. This involves inducing the chemotaxis of Th2 cells and the alternative activation of macrophages. In the meantime, genes including SELENOP,PDK4,and ALDH1A1 work in concert to coordinate antioxidant defense mechanisms and metabolic reprogramming. This assists in preserving cell homeostasis under conditions of oxidative stress and hypoxia while also supplying energy. Moreover, APOE, MRC1, and CHI3L1 are implicated in lipid metabolism and tissue repair processes. Additionally, SLC40A1,FTL, and GPNMB are involved in mediating iron homeostasis and the remodeling of the extracellular matrix (38, 39). This particular subset also secretes proteinases and molecules related to lysosomes, such as CTSD,CTSC,CTSB,CTSZ, and CTSS, which serve to govern cell interactions and tissue remodeling. Collectively, the expression profile of Macro_C1QC mirrors the intricate interplay among macrophages, tumor cells, and other immune cells (**Figure 4c**, **Supplementary Table 11**). It performs a pivotal role in driving tumor progression, facilitating immune evasion, and contributing to the remodeling of the tumor microenvironment.

Among the various findings, the enrichment of Macro_SPP1 in metastatic lesions holds the most clinical significance.Macro_SPP1 secretes chemokines such as CCL3L1,CCL4,and CCL3,which effectively recruit immune cells like T cells and eosinophils.CX3CR1 facilitates the precise localization and monitoring of tumor tissue by these macrophages (40). The expression of C3 allows their involvement in complement activation within the tumor microenvironment, thereby influencing tumor cell lysis and immune cell activation.ALOX5AP and LTC4S participate in arachidonic acid metabolism, modulating inflammatory responses and immune cell functions. The expression of heat-shock protein-related genes such as HSPA6,HSPA1B, and HSPA1A equips this macrophage subset to cope with the stressful tumor microenvironment and play a role in immune regulation. Cell-skeleton-related genes like CORO1A influence their shape, movement, and phagocytic function, thereby altering their motility and functional state within the tumor microenvironment. Immunoglobulin Fc receptor-related genes such as FCGR2A, FCGR3A, and FCGR1A mediate the binding of macrophages to immune complexes, regulating phagocytosis and cytokine secretion. Genes like TREM2 and TGFBR1 are involved in regulating the immune response, activation status, and functional polarization of macrophages (41, 42). The expression of genes such as SPP1,OLFM3,HERPUD1,and CSF1R is related to cell adhesion, migration, proliferation, differentiation, as well as the development and survival of macrophages, thus affecting tumor cell invasion, metastasis, and the structural and functional shaping of the microenvironment (**Figure 4c**, **Supplementary Table 12**). Macrophages are highly plastic, showing phase-specific activation during progression of BC (43, 44). We observed a mix of pro-inflammatory and pro-resolution macrophages, with varying proportions and functions. The relative proportion of pro-inflammatory macrophages (Macro_IL1B) increased transiently in Normal. In contrast, pro-resolution macrophages (Macro_C1QC) were downregulated (**Supplementary Figures 2a, b**).

Previous studies have documented macrophages differentiating from monocytes in primary tumors (45). We proceeded to investigate whether the macrophage differentiation trajectory remains consistent across different groups. By employing trajectory analysis on the identified monocytes and macrophage subtypes, we delineated the myeloid cell differentiation trajectories in BC (**Figure 4e**, **Supplementary Figure 2d**). The findings demonstrated that macrophages in normal, ER,HER2, and ER_LN subtypes predominantly differentiate from monocytes, and distinct myeloid subtypes with unique differentiation trajectories were enriched.

We conducted an in-depth analysis of pseudotime genes among the ER,HER2, and ER_LN groups across five myeloid subtypes. We identified 156 genes common to all three tumor subtypes.644 genes were found in HER2, while ER contained 48 genes and ER_LN 29 genes (**Supplementary Figure 2c**). Monocle2-based trajectory analysis showed marked divergence in gene regulatory programs between myeloid subsets, and GO enrichment revealed distinct biological processes underlying each subtype’s adaptation to the tumor micro environment (TME).

In ER, myeloid-derived cells showed enrichment for hydrogen peroxide response and intracellular pH reduction. This suggests a heightened adaptation to oxidative stress and metabolic reprogramming, which may be related to endocrine therapy resistance. The simultaneous enrichment for apoptotic cell clearance implies that myeloid cells play a role in immunosuppression through efferocytosis. This process may maintain tumor tolerance by clearing cellular debris and inhibiting pro-inflammatory signals. These findings are consistent with the immunologically “cold” nature of ER tumors and may explain their reduced responsiveness to immune checkpoint inhibitors (46) (**Supplementary Figures 3d, e**).

In HER2, myeloid populations displayed distinctive associations with hypoxia responses, enhanced cell migration regulation, and widespread activation of apoptotic processes. Hypoxia-linked pathways likely mirror the aggressive angiogenic dysregulation typical of HER2 tumors, while heightened migratory signaling might bolster the infiltration of myeloid-derived suppressor cells (MDSCs) and the formation of pre-metastatic niches. Paradoxically, apoptotic signaling could indicate a dual mechanism where tumor control and immune evasion coexist, possibly through the apoptosis of cytotoxic immune cells dampening anti-tumor responses (**Supplementary Figures 3d, f**).

Interestingly, in ER_LN, myeloid cells showed specialization in cellular senescence-related pathways, mammary gland epithelial cell proliferation, and angiogenesis. Cellular senescence-associated phenotypes could drive the epithelial-mesenchymal transition and stromal remodeling that support metastatic outgrowth (47, 48). The concurrent activation of proliferative and angiogenic pathways underlines the myeloid-TME crosstalk in sustaining metastatic colonization, perhaps via direct interaction with EC (**Supplementary Figures 3d, g**).

Our study uncovers a previously underrecognized dimension of myeloid plasticity that is closely aligned with the specific oncogenic drivers of different tumor subtypes.ER myeloid cells show metabolic symbiosis and immune silencing features. HER2 subsets are more inclined to hypoxic adaptation and inaccessibility mot. ER_LN populations take on pro-senescent and angiogenic programs to promote metastasis. Targeting these subtype-specific pathways with therapies like hydrogen peroxide scavengers for ER, hypoxia-inducible factor inhibitors for HER2, or senescence-disrupting agents for ER_LN may disrupt key myeloid-mediated support networks. This could provide novel combination strategies to counter immune evasion and metastasis.

### Heterogeneity of T cell subsets in BC

**Figure 5:**
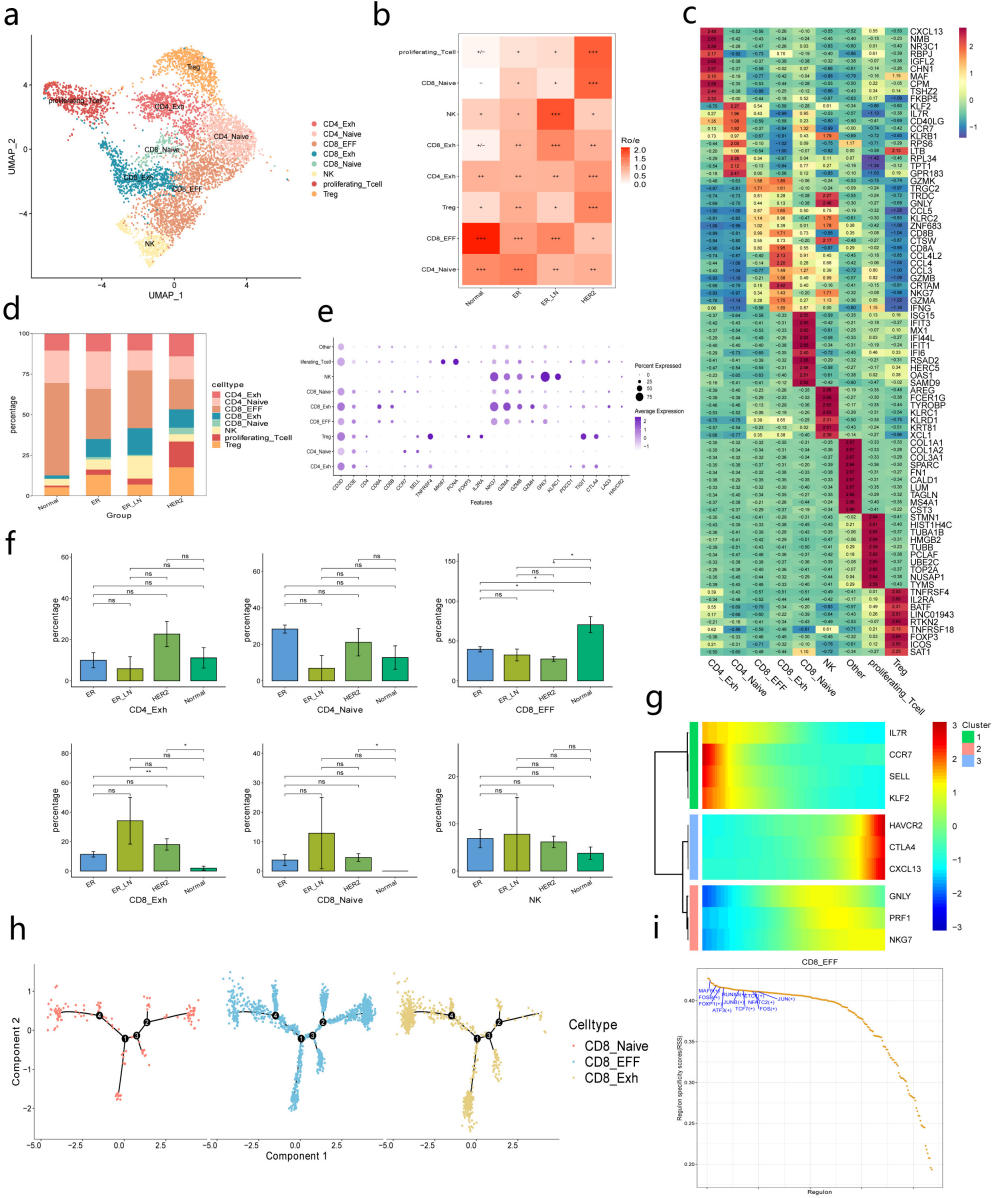
Characterization of T/NK subsets. **(a)** UMAP-plot showing cell types of T/NK cell subsets. **(b)** Heatmap showing the BC suntypes distribution of each major T/NK cell type by Ro/e analysis.[Ro/e > 1]:+++,[0.8 < Ro/e <= 1]:++,[0.2 <= Ro/e <= 0.8]:+,[0 < Ro/e < 0.2]: +/-,[Ro/e = 0]:-. **(c)** Heatmap displaying the expression of the top 10 differentially expressed genes of major T/NK cell type. **(d)** Bar chart showing the relative proportion of each T/NK subset in different BC subtypes. **(e)** The expression levels of selected functional genes in T cell subsets. **(f)** Bar plots showing the differences in the proportions of major T cell subtypes between four BC subtypes. Statistical testing was performed by a two-sided Wilcoxon test. Data are presented as mean values+/− SD. **(g)** The expression patterns of genes associated with the pseudotime. Colors on heatmap indicate the genes expression. Color from red to blue indicates a high to low expression. **(h)** Developmental trajectory of CD8_Naive,CD8_EFF and CD8_Exh subtypes in the BC. Colors of cell indicate cell type marked by legend. **(i)** Scatter plots of TF regulons based on specificity Z scores in CD8_EFF.

To elucidate the inherent structure and potential functional subtypes of lymphoid cells, the T cell populations underwent further subdivision into 12 sub-clusters. This included 3 clusters for CD4+ T cells,4 clusters for CD8+ T cells,1 cluster for proliferating cells,1 cluster for NK cells, and 3 undefined clusters (**Figure 5a**, **Supplementary Figures 4a, b**). Each cluster demonstrated specific expression of unique signature genes (**Figure 5c**).

Two subtypes of inhibitory CD4+ T cells (Treg and CD4-Exh) were identified. Treg was distinguished by specific FOXP3 expression, while CD4-Exh exhibited high levels of CTLA4,PDCD1,and CXCL13.CD4_Naive cells were recognized by SELL and CCR7 expression (**Figure 5e**). No clear correlation was observed between the frequency of CD4-Exh and CD4-Naive cells across cancer subtypes (**Figures 5b, d, e**), suggesting that the CD4+T cell composition remains relatively consistent in ER,HER2,and ER_LN subtypes.

A substantial group of CD8+ T cells was also detected (**Figures 5a, e**), which could be categorized into three subtypes:CD8_Naive,CD8_EFF,and CD8_Exh.CD8_Naive cells showed high expression of ISG15 and IFIT3,with no significant changes in abundance between groups.CD8_EFF cells were enriched in normal tissues, displaying higher expression of GZMK, GNLY, and KLRC2.The proportion of the exhausted T cell cluster CD8_Exh,marked by high PDCD1,LAG3 and HAVCR2 expression, was elevated in tumors (49) (**Figures 5b–d**). These specific changes in cell subset distribution imply that the three metastatic groups may possess unique immune microenvironment characteristics, which in turn influence tumor occurrence, development, and immune response processes.

Further exploration of the signature genes of the subset with marked fluctuations in cell frequency reveal that Treg cells show elevated expression of a range of genes intimately associated with immunosuppressive functions. Take FOXP3,the pivotal transcription factor, for example. As the central regulatory element of Treg cells, FOXP3 is of utmost importance for preserving the development and functionality of Treg cells (50). Simultaneously, the high expression of immune checkpoint molecules like CTLA4 and TIGIT implies that Treg cells could establish an immunosuppressive state within the tumor microenvironment by suppressing the activation and proliferation of effector T cells, thereby facilitating tumor immune evasion. Notably, the coordinated upregulation of tumor necrosis factor receptor superfamily members (TNFRSF4,TNFRSF9,TNFRSF18) suggests enhanced immunosuppressive activity via TNFR signaling pathways (51). In addition, the expression of genes such as IL2RA and BATF indicates that Treg cells play a crucial role in cell survival, metabolism, and the immune regulatory network (52). They jointly contribute to the construction of the immunosuppressive microenvironment in BC (**Figure 5c**, **Supplementary Table 13**).

Regarding CD8_Exh cells, their characteristic gene profiles exhibit typical molecular features of T-cell exhaustion. The high expression of inhibitory receptors such as LAG3,KLRG1, and TIM3 implies that the abnormal expression of these molecules is closely related to T-cell functional failure and the weakening of anti-tumor immune responses (53). Although CD8_Exh cells still express some cytotoxic-related genes like GZMB and NKG7, their overall function is in a hyporesponsive state and may not effectively kill tumor cells. Moreover, the expression of chemokines such as CCL3,CCL4, and CCL5 may influence T cell recruitment and localization, further exacerbating the immunosuppressive state in the tumor microenvironment (54). The aberrant activation of transcription factors (ZEB2,RUNX3) and mitochondrial dysfunction-associated genes (MT-ND3) indicates that metabolic reprogramming drives exhaustion. Remarkably, the shared TNFRSF9 (4-1BB) signaling in both CD8_Exh and Tregs highlights dual immunosuppressive mechanisms that are amenable to co-stimulatory pathway targeting (55, 56) (**Figure 5c**, **Supplementary Table 14**).

The decline in the proportion of CD8_EFF cells and the alterations in their signature genes directly reflect the weakening of anti-tumor effector functions (**Figures 5b, d**). The signature genes of CD8_EFF cells are enriched in pathways related to cytotoxicity, effector functions, and immune activation. The expression of genes such as GZMK, GNLY, and CCL5 is closely associated with T cell-mediated killing and immune regulatory functions (57). The decrease in their proportion means that the body’s effector immune response against tumors is suppressed, enabling tumor cells to evade clearance and thereby promoting BC progression and metastasis (**Figure 5c**, **Supplementary Table 15**).

In our subsequent exploration of the dynamic immune states and cell transitions within infiltrated CD8+ T cells, we leveraged Monocle2 to infer state trajectories. The results indicated that CD8_Naive cells were positioned at the trajectory’s origin, while CD8_Exh cells resided at the terminal state (**Figure 5h**, **Supplementary Figure 4c**). Next, we delved into the transcriptional changes linked to transitional states, discerning that CD8+ T cell clusters could be divided into three distinct phases.

Phase 1 cells were defined by upregulated SELL,IL7R,KLF2,and CCR7,alongside low GZMA, GZMB, and GZMH expression, imparting the lowest cytotoxic capacity. In phase 2,cells exhibited maximal GNLY,NKG7,and PRF1 expression, aligning with the phenotype of classical cytotoxic T cells. Phase 3 cells were marked by heightened expression of T cell exhaustion-related genes, such as HAVCR2,CXCL13,CTLA4,and TIGIT, along with mitotic pathway genes, further solidifying their exhausted state (**Figure 5g**, **Supplementary Figure 3d**).

In pursuit of transcription factors (TFs)associated with CD8_EFF cell transition directions, we conducted gene regulatory network analysis using pySCENIC. We unveiled a series of posedotime regulons co-expressed in CD8_EFF and CD8_Exh,including RUNX3,ATF3,MAFF and FOSB.Notably,FOXP1,JUNB,TCF7,and JUN were predominantly present in CD8_EFF cells, signifying their early effector and memory-activated T cell states and their role in preventing CD8+ T cell exhaustion (**Figure 5i**). Conversely,CD8_Exh cells were distinguished by PRDM1,CREM,and BHLHE40 (**Supplementary Figure 3e**). PRDM1 possibly facilitates tumor immune evasion by elevating PD-L1 expression, thereby blunting anti-tumor effects (58, 59). The ADRB1-camp-CREM signaling axis in CD8+ T cells has been documented, indicating that Adrb1 inhibits CD8+ TCR activity and fosters exhaustion via cAMP-CREM signaling (60). BHLHE40 also plays a pivotal role in T cell exhaustion. Together, these results indicated that BC were enriched with infiltrated T cell subtypes with distinct status (61).

### Constructing an EC subtypes-based regulatory network for BC

**Figure 6:**
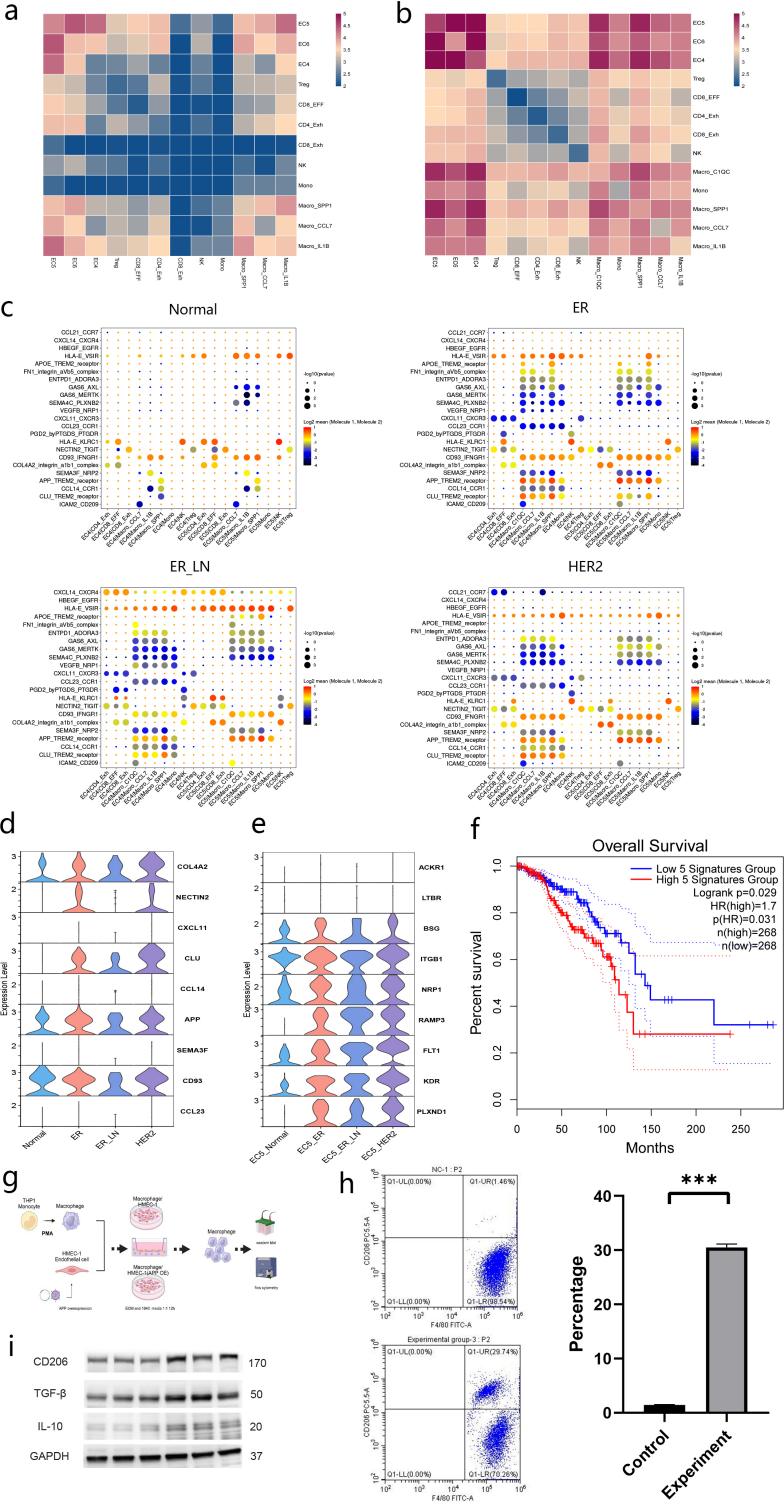
Development of an EC-based regulatory network for BC. **(a)** Heatmap showing the interaction intensity among major cell subsets from Normal. **(b)** Heatmap showing the interaction intensity among major cell subsets from ER. **(c)** The interaction of selected T cell and Myeloid subsets with EC5 in individuals with Normal (leftmost),ER (second from the left),ER_LN (second from the riguht)and HER2 (rightmost).the dot size and color indicate the interaction strength. **(d, e)** Violin plot showing the expression levels of indicated ligand genes among the indicated groups. Colors on the columns indicate groups marked by x-axis. **(f)** Kaplan–Meier plots showing the survival probability of BC patients with high and low co-expression levels of CD47 and SIRPA.Red line indicates patients expressed higher FN1,ITGB1,VEGFA,KDR and NRP1 co-expression. blue line indicates patients expressed lower genes above co-expression. P values were calculated by log-rank test. Time (x-axis) is represented in months. **(g)** The cell interaction experimental workflow for EC and macrophages. **(h)** Flow cytometric quantification of CXCL8-producing immune cell subsets in cervical cancer tissues. **(i)** TGFB1,IL10 and MRC1 protein levels were assessed by Western blot.

Cellular interactions between EC and immune cells hold potential therapeutic significance, particularly in immunotherapy, as EC serve as a vital interface with the immune system (7, 8).However,previous scRNA-seq studies in BC have not extensively examined this aspect (11, 12). Our study identified EC4 and EC5 as two distinct endothelial subclusters uniquely present in tumor tissues, which are significantly associated with patient prognosis. We further investigated the cell-cell interactions between Endothelial (EC4&EC5) and immune cells using CellPhoneDB (62). Compared with healthy states, EC exhibited enhanced interactions with immune cells in the tumor microenvironment (**Figures 6a, b**, **Supplementary Figure 3f, g**).Focused on the EC4 endothelial cell cluster, it displayed distinct intercellular communication patterns with CD8+ T cells between the two groups. In the tumor group, three receptor-ligand pairs were found to be enriched, namely the COL4A2-integrin α1β1 complex,NECTIN2-TIGIT and CXCL11-CXCR3.The COL4A2-integrin α1β1 complex may influence the adhesion and migration of CD8+ T cells, facilitating their migration toward the tumor site and promoting anti-tumor immune responses. TIGIT delivers inhibitory signals to CD8+ T cells, which may somewhat weaken their anti-tumor activity. Notably,NECTIN2-TIGIT was mainly enriched in the interactions between EC4 and CD4_Exh as well as CD8_Exh cells, rather than CD8+EFF cells. This suggests a selective interaction pattern of NECTIN2-TIGIT in the tumor setting (63). CD4_Exh and CD8_Exh cells, which often suffer functional impairments due to chronic antigen stimulation, may engage in specific communication with EC4 cells via the NECTIN2-TIGIT axis, potentially affecting the tumor-associated immunosuppressive microenvironment. The CXCL11-CXCR3 signaling pathway plays a crucial role in the chemotaxis of CD8+ T cells (64), promoting their migration to the tumor site and enhancing their infiltration (**Figure 6c**).

In the tumor group, EC4 EC demonstrated unique intercellular communication traits with macrophages. Several receptor-ligand pairs were enriched, Including CLU-TREM2,CCL14-CCR1,APP-TREM2,SEMA3F-NRP2,CD93-IFNGR1 and CCL23-CCR1.The CLU-TREM2 interaction might modulate the activation state of macrophages and promote their polarization toward a tumor-associated macrophage (TAM) phenotype. TAMs frequently display immunosuppressive functions that can inhibit the anti-tumor immune response (65). Likewise,the APP-TREM2 interaction could also influence macrophage activation and function.CCL14-CCR1 and CCL23-CCR1 signaling may impact macrophage migration and recruitment.NRP2 in myeloid cells is an inflammation-responsive protein that is rapidly induced under inflammatory conditions.SEMA3F-NRP2 signaling is implicated in regulating cell migration and inflammatory responses (66). IFNGR1 is the receptor for interferon-γ (IFN-γ).The binding of CD93 to IFNGR1 may affect macrophage responsiveness to IFN-γ, influencing their activation and polarization (**Figures 6c, d**). TREM2-positive macrophages are enriched in the BC microenvironment and are involved in immune suppression and tumor progression. However, the research has focused on the macrophages themselves, without involving the upstream APP-positive vascular EC.

To elucidate the role of APP-positive EC (APP+ EC) in driving macrophage polarization towards an M2-like phenotype, we established a co-culture system comprising APP-overexpressing EC and macrophages. Flow cytometry analyses revealed that APP+ EC significantly promoted the differentiation of macrophages into an M2-like phenotype, as evidenced by the upregulation of canonical M2 markers, including TGFB1,IL10,and MRC1 (**Figures 6g–i**). These findings demonstrate that APP+ EC foster immunosuppression by polarizing macrophages into M2-like cells, thereby highlighting the necessity to explore their broader immunoregulatory functions.

The initiation of the innate immune response is pivotal for angiogenesis and tissue repair. Subsequently, we investigated the role of immune cells in the functional alterations of EC5 cells (67). In tumor tissues,EC5 cells exhibited selective enrichment of ligand-receptor (L-R)pairs with CD8+ T cells (**Figure 6c**). For instance, tumor-infiltrating CD8+ T cells secreted CCL5,which bound to ACKR1 (atypical chemokine receptor 1)on EC5 cells.ACKR1,a scavenger receptor for inflammatory chemokines, may sequester CCL5 to attenuate T cell recruitment (68). Paradoxically,this process may facilitate immune evasion. Additionally, the lymphotoxin-beta (LTB)-LTBR axis suggests that EC5 cells may orchestrate the formation of tertiary lymphoid structures (TLS)within tumors. However, concurrent TGFB1_TGFBR3 signaling implies counterbalancing immunosuppressive effects (69). Specifically,TGFBR3 (betaglycan) expressed by EC5 cells captures TGFB1 from CD8+ T cells, potentially activating latent TGF-β signaling in adjacent fibroblasts or regulatory T cells to promote stromal desmoplasia. Furthermore, the interaction between cyclophilin A (PPIA)and BSG (CD147), which is known to drive matrix metalloproteinase (MMP)production, may facilitate EC5-dependent vascular co-option and metastasis.

Macrophages and EC5 exhibit enrichment of eight receptor-ligand pairs, including FN1_integrin,VEGFA_NRP1,ADM_RAMP3,COL6A2_integrin_α1β1_complex,VEGFA_FLT1,VEGFA_KDR,CCL2_ACKR1,SPP1_integrin_α9β1_complex, SEMA4A_PLXND.Within the tumor microenvironment, the activation of PPARG on EC may modulate their metabolic state and function, potentially influencing vascular permeability and the tumor’s nutrient supply. Adrenomedullin (ADM)is a multifunctional peptide with vasodilatory, angiogenic, and anti-apoptotic properties. Its interaction with RAMP3 on EC5 may promote endothelial cell survival and angiogenesis (70).In the context of tumors, this interaction could contribute to the formation of new blood vessels, thereby providing the tumor with essential nutrients and oxygen. The binding of collagen type VI alpha 2 (COL6A2) from macrophages to integrin α1β1 on EC5 may alter the adhesion properties and migratory behavior of EC. This interaction could influence the stability of blood vessels and the infiltration of immune cells into the tumor. The interaction between vascular endothelial growth factor A (VEGFA)from macrophages and FLT1/KDR on EC5 is likely to play a substantial role in promoting angiogenesis. VEGFA signaling through these receptors activates downstream pathways that drive endothelial cell proliferation, migration, and tube formation, all of which are critical for new blood vessel formation.Additionally,FN1-integrin,VEGFA_KDR and VEGFA-NRP1 interactions could serve as potential signals associated with poor prognosis for renal cell carcinoma (RCC)bone metastasis (71, 72) (**Figures 6c ,e, f**).

These interactions between macrophages and EC5, mediated by the identified receptor-ligand pairs, underscore the complex and multifaceted communication between immune cells and EC in the BC tumor microenvironment. The activation of these signaling pathways may lead to significant changes in EC5 function, including alterations in angiogenesis, vascular permeability, cell adhesion, and immune cell trafficking. These changes are likely to contribute to the progression of BC by facilitating tumor growth, metastasis, and immune evasion. Further studies are required to fully elucidate the mechanisms underlying these interactions and their functional consequences, which may provide valuable insights into potential therapeutic targets for BC treatment.

## Materials and methods

### Data acquisition

Transcriptomic and clinical data for BC were sourced from the Gene Expression Profiling Interactive Analysis (GEPIA2)platform (http://gepia2.cancer-pku.cn,accessed Aperal 13,2025). The clinical variables incorporated in this study included survival time, survival status, age, gender, and TNM staging information. Additionally, the scRNA-seq dataset GSE161529 was obtained from the Gene Expression Omnibus (GEO)database (https://www.ncbi.nlm.nih.gov/geo/query/acc.cgi?acc=GSE161529,accessed August 13,2024).The spatial transcriptome data are derived from Spatial TME’s BC_Visium_FreshFrozen_WholeTranscriptome_10x (https://www.spatialtme.yelab.site/#!/browse/BC).

This dataset encompasses profiles of 4 HER2 tumors,4 ER tumors, and 4 lymph node metastases associated with ER tumors. It also includes comprehensive profiles of mammary gland cells from 4 individuals without BC (**Supplementary Table 16**).

### Quality control, clustering, normalization identification of the major cell types and their subtypes

Single-cell RNA-sequencing expression profiles were generated for 16 samples using the 10x Genomics Chromium platform and sequenced on an Illumina NextSeq 500 instrument (**Figure 1a**, **Supplementary Table 1**). Gene-wise read counts were obtained for all samples using Cell Ranger version 3.0.2 (https://support.10xgenomics.com). Specifically,Illumina base call files (BCLs)were demultiplexed into FASTQ files using the “cellranger mkfastq” command, followed by the generation of gene-wise read counts with “cellranger count,” utilizing the Cell Ranger human GRCh38 reference version 3.0.0.Default settings were applied for all parameters, with the exception of file locations and memory allocation. For each biological sample, the output from the Cell Ranger directory “outs/filtered_feature_bc_matrix” was utilized, which comprises three files: the count matrix in matrix market (mtx.gz)format, the cellular barcodes (barcodes.tsv.gz),and the gene identifiers (features.tsv.gz).While the gene identifiers are consistent across all samples, the count matrix and barcode files are specific to each sample.

Subsequently, quality control procedures were implemented for each sample using the Seurat package. This involved filtering out cells based on criteria such as the number of detected genes, total unique molecular identifier (UMI)counts, and the proportion of total UMI counts derived from mitochondrial genes. Following quality control, data normalization was performed using the “NormalizeData” function with default parameters. To identify the most variable genes, the “FindVariableFeatures” function was applied. To ensure comparability across different single-cell RNA-sequencing samples within the same study, scale normalization was conducted using the “ScaleData” function to harmonize total UMI counts. Integration of the data was explored using the Harmony method, implemented with the “RunHarmony” function and default settings. Principal component analysis and uniform manifold approximation and projection (UMAP)were subsequently performed using the “RunPCA” and “RunUMAP” functions, respectively. Cells were then clustered into distinct groups in the embedding space using the “FindClusters” function. Ten major cell types were identified: T/NK cells (marked by CD3D,CD3E,and KLRC1),myeloid cells (marked by CD14,FCGR3A,and LYZ),B cells (marked by CD79A and MS4A1),EC (marked by PVALB and PECAM1),fibroblasts (marked by COL1A1 and COL1A2),and epithelial cells (marked by EPCAM,KRT18,and VEGFA).

To further resolve subclusters within each major cell type, cells belonging to each cell type were re-analyzed separately. This process included quality control, dimensionality reduction, and clustering using an unsupervised graph-based clustering algorithm. Subclusters were annotated to specific cell subtypes based on subcluster-specific marker genes, as detailed in the corresponding figures and **Supplementary Data 1**.

### Trajectory analysis

To elucidate the cell lineage trajectories of CD8+ T cells and macrophages, we employed Monocle2 (Qiu et al., 2017).Initially, we utilized the “relative2abs” function within Monocle2 to transform transcripts per million (TPM)values into normalized mRNA counts. Subsequently, we constructed a Monocle2 object, specifying the parameter “expressionFamily = negbinomial. size” in accordance with the Monocle2 tutorial. To identify differentially expressed genes (DEGs)within each cluster, we applied the “differential GeneTest” function. Genes with a q-value threshold of less than 1e-5 were selected to order the cells along the pseudotime trajectory. Following the construction of the cell lineage trajectories, we further detected differentially expressed genes along the pseudotime continuum using the “differentialGeneTest” function.

### Tissue distribution of clusters

To quantify the tissue preference of each cell type or subtype across normal, primary, and lymph-node metastatic BC tissues, we calculated the ratio of observed to expected cell numbers (Ro/e)for each cell type or subtype. The expected cell numbers for each combination of cell type or subtype and tissue were derived from the chi-square test. A Ro/e ratio greater than 1 indicated that a particular cell type or subtype was enriched in a specific tissue.

### Polarization state and functional phenotypes analysis of macrophages subtypes

To further elucidate the distinct M1/M2 polarization states and functional phenotypes of macrophage subtypes, we assessed gene sets associated with M1 and M2 phenotypes (**Supplementary Table 3**) by evaluating the mean expression levels of cells within each macrophage subtype. To quantify the expression patterns of these gene sets, we employed the “addModuleScore” function. This function integrates the normalized expression values of all genes within a specific gene set, thereby generating a module score. These module scores provide a comprehensive representation of the key functional gene components relevant to the biological processes under investigation.

### Cell communication analysis

Cell-cell interactions were examined using the CellPhoneDB Python package [54].To manage computational complexity while retaining representation of diverse cell types, we performed downsampling by randomly selecting 1,000 cells from each cell type. The interaction strength was determined based on the co-expression of receptors and ligands between different cell types. Specifically, only receptors and ligands expressed in more than 30% of the cells within a given cluster were included in the analysis. To assess the significance of these interactions, we permuted the cluster labels of all cells 1,000 times to calculate the P-value for the likelihood of each paired interaction. Only interactions with a P-value below 0.05 were deemed significant.

To identify distinct paired interactions between ECs and other cell types, we utilized the “FindAllMarkers” function to detect differentially expressed genes. Genes were filtered based on a log fold change (LogFC) threshold greater than 0.5 and a minimum percentage of expressing cells (min.pct)greater than 0.25. These criteria ensured the robust identification of relevant gene pairs involved in cell-cell communication.

### Transcription factor analysis

To identify activated transcription factor (TF)regulons in each CD8+ T cell subset, we employed the SCENIC pipeline [36]. The pySCENIC package (version 0.11.2)was utilized, with the raw count matrix serving as the input data. Specifically, regulons were initially identified using the RcisTarget algorithm, which detects potential TF binding sites in the regulatory regions of genes. Subsequently, the co-expression network was constructed using the GRNBoost2 algorithm, which infers gene regulatory networks based on mutual information. Finally, the activity of each regulon in individual cells was quantified using the AUCell algorithm, which assesses the enrichment of regulon target genes in single cells.

### Pathway analysis

Differentially expressed genes (DEGs)for each cell subtype were identified using the “FindMarker ()” function from the Seurat package. The criteria for significance were set as an absolute fold change (|FC|)greater than 0.25 and an adjusted P-value (adj.P-val) less than 0.05.To further explore the biological functions associated with these DEGs, we performed pathway analysis using the MetaScape webtool (https://metascape.org/gp/index.html).

Gene Ontology (GO)terms were utilized to annotate the functional enrichment of DEGs. The false discovery rate (FDR)was calculated using the Benjamini–Hochberg correction procedure to account for multiple hypothesis testing. Among the top 100 enriched GO terms across different cell types, five to ten GO terms or pathways were selected for visualization. Heatmaps were generated using the heatmap package (version 1.0.12),and ggplot2 was employed for additional graphical representations.

### Spatial transcriptomic analysis

To elucidate the spatial distribution of gene expression profiles associated with distinct cell types, we leveragedSpatial TME (https://www.spatialtme.yelab.site/#!/analysis/Spatial_Gene_Expression), an integrative database specifically designed to characterize the tumor microenvironment through spatial transcriptomics. This resource enabled us to visualize the spatial expression patterns of the selected genes or gene sets corresponding to specific cell types. Cancer type:BRCA.DOI: https://www.10xgenomics.com/cn/resources/datasets/human-breast-cancer-visium-fresh-frozen-whole-transcriptome-1-standard.

### The Cancer Genome Atlas data analysis

To investigate the associations between selected genes and patient survival outcomes, we utilized the Cancer Genome Atlas Breast Invasive Carcinoma (TCGA-BC)datasets. We employed GEPIA, a comprehensive web-based platform designed for cancer and normal gene expression profiling and interactive analyses, to assess the impact of individual genes or gene signatures on patient survival. Kaplan–Meier survival curves were subsequently generated using this platform to visually represent these correlations.

### Immunohistochemistry

The parafn-embedded sections of BC tumor and adjacent tissues were deparafnized and then, treated with EDTA Antigen Retrieval Solution(Dowobio China DW2012) for 2 min with high-pressure treatment, and endogenous peroxidase and avidin activities were blocked using an endogenous peroxidase blocking buffer (Dowobio, China, DW 2177).Then,3% BSA was used to block the sections at room temperature for 30 min. Next, the sections were incubated overnight at 4 °C with anti-FBLIM1 antibody (1:500,PA5-55,236,Invitrogen).Subsequently, the secondary antibody was added and incubated at room temperature for 60 min. Finally, the sections were staining using DAB kit (Dowobio,China,DW2033)and hematoxylin.

### Cell culture and differentiation

The human monocytic leukemia cell line THP-1 was maintained in RPMI-1640 medium supplemented with 10% fetal bovine serum (FBS)and 1% penicillin-streptomycin. To differentiate THP-1 monocytes into macrophages, cells were treated with 100 ng/mL phorbol 12-myristate 13-acetate (PMA)for 48 hours. Following differentiation, the medium was replaced with fresh PMA-free complete medium, and the cells were rested for an additional 24 hours before subsequent experiments. Human Microvascular Endothelial Cell line-1 (HMEC-1)were cultured in endothelial cell growth medium.

### Lentiviral transduction of EC

EC were seeded onto the upper chamber of a Transwell insert (0.4 μm) and allowed to adhere overnight. Upon reaching 50-60% confluence, the cells were transduced with lentiviral particles carrying the gene of interest (GOI)or an empty vector control at a predetermined multiplicity of infection (MOI)in the presence of 8 μg/mL polybrene. After 24 hours, the virus-containing medium was replaced with fresh growth medium.

### Co-culture system and experimental setup

After 48–72 hours of transduction to allow for sufficient gene expression, the Transwell inserts containing the transduced EC were carefully transferred and placed into the wells of a 12-well plate containing the differentiated THP-1-derived macrophages. The two cell types were co-cultured for 12 hours in a shared culture medium to allow for paracrine communication.

## Discussion

The present study delineates a high-resolution landscape encompassing human primary BC (ER and HER2 subtypes)alongside lymph-node metastases of the ER subtype, while delving into the associated molecular mechanisms underpinning BC. Through the utilization of scRNA-seq data, a comparative analysis has been conducted regarding the transcriptomic profiles of EC derived from HER2 tumors and ER_LN, set against the backdrop of ER primary tumors. At present, the dominant therapeutic approach for most human cancers involves an indiscriminate anti-angiogenic strategy, which predominantly targets the VEGF signaling pathway (14, 17). However,it is important to note that EC (ECs)exhibit a significant degree of phenotypic heterogeneity, not only when comparing different tissues but also the across vascular tree within a single tissue. Nevertheless, consistent with findings from other single-cell investigations of human malignancies (14, 73), actively dividing EC (ECs)were not identified within our scRNA-seq data derived from human breast tumors. Given that the ER subtype represents the most common form of BC, prior investigations have employed scRNA-seq technology to identify the cell of origin and uncover transcriptomic differences among various cell types (74). The study conducted by Peter et al. revealed that, in comparison to pECs, TECs demonstrated enrichment of gene sets implicated in ECM remodeling and oxidative phosphorylation, while concurrently exhibiting reduced expression levels of gene sets associated with lipid metabolism (31). Within our investigation, it was observed that EC in primary tumors displayed a greater propensity for shaping the tumor microenvironment and promoting angiogenesis when contrasted with normal EC.

To date, the functional heterogeneity of EC across different BC tumor subtypes has not been comprehensively elucidated (31).Our findings demonstrate that the distribution and function of EC in normal tissue and the three tumor subtypes exhibit significant distinctions. These three tumor subtypes are enriched in endothelial subclasses that are involved in immune signaling and matrix formation, which play a crucial role in modulating the immune microenvironment. Furthermore, EC from different tumor subtypes exhibit functional specificity. When compared to ER,EC from ER_LN display higher expression levels of genes related to immune response and cell proliferation/differentiation. In the case of HER2 EC, there are alterations in functions associated with immunosuppression, angiogenesis, and mitochondrial reorganization. Additionally, we have identified conserved endothelial subtype marker genes across different tumor subtypes.

A variety of immune cells, such as neutrophils, dendritic cells, macrophages, and lymphocytes (including B cells and T cells),are recognized to stimulate vessel sprouting through the production of pro-angiogenic molecules. Moreover, perivascular macrophages are known to facilitate the fusion of adjacent vessel sprouts, thereby contributing to the formation of perfused vessels (75, 76). Given the relative lack of in-depth documentation concerning EC-immune cell interactomes, our RLI dataset provides a valuable opportunity to explore the multitude of potential molecular signals that underlie these intercellular communications.

Our receptor-ligand interactome predictions have not only identified known interactions—thereby serving to validate our methodology—but have also uncovered previously unrecognized or poorly characterized interactions between ECs and immune cells. Some of these interactions suggest that ECs may fulfill different immunomodulatory roles across various BC subtypes (77, 78). For instance, we have predicted specific interactions between tumor-enriched angiogenic ECs and subtypes of CD8+ T cells or Macrophage cells, which may hold implications for tumor angiogenesis in different subtypes of BC. Collectively, these predictions (i)raise questions regarding the potential regulatory role of the EC/immune cell interactome in tumor immunity and vessel sprouting in BC, possibly through previously unknown interactions with CD8+ T cells or Macrophage subtypes, among others; (ii)provide an extensive resource to encourage future functional studies aimed at characterizing the immunomodulatory functions of ECs, as well as the reciprocal communication between ECs and immune cells; and (iii)address the gap in the current understanding of the absence of an endothelial and immune cell interaction network among different subtypes of BC tumors.

We recognize several limitations in our study. Initially, the biological functions of the EC subclusters identified through marker gene analysis are hypothetical and need experimental validation to confirm their roles. Secondly, since tissue dissociation did not specifically enrich EC, their abundance in the samples might be inadequate for comprehensive analysis. For further in-depth study of EC,EC should be enriched, although doing so will lead to changes in the proportion of other cell types. A limitation of our study is that mature adipocytes are absent from our transcriptomic analyses because scRNAseq is not amenable to sequencing this particular cell type (79).Thirdly,while we profiled metabolic pathways at the transcriptional level, this approach does not fully capture the complexity of metabolic fluxes, metabolite concentrations, and enzyme activities at the protein level. That being said, prior studies have indicated that gene expression signatures can partially predict changes in metabolic fluxes within ECs (80, 81). Fourthly, the RLI pairs we identified are based on predictive models and require additional experimental validation to confirm their biological relevance (82, 83). Finally, the associative correlations observed in our retrospective study need to be validated through prospective trials to establish their clinical significance.

Despite these limitations, our study offers a comprehensive transcriptomic resource on EC heterogeneity in peri-tumoral and malignant breast tissue. It also provides a predicted interactome of stromal cells in BC, which can serve as a foundation for future research into their functional roles. Furthermore, our findings highlight key ECs that could be prioritized for functional and biomarker studies in the future.

## Conclusion

In summary, our study presents a systematic delineation of the cellular landscape in BC, shedding light on the intratumoral heterogeneity inherent in primary tumors and lymph-node metastases of BC. Through rigorous analysis, we have pinpointed specific cellular subsets and molecular characteristics that are enriched within the diverse microenvironments of BC subtypes. Furthermore, our investigation into developmental trajectories and cell-cell interactions has unveiled particular subtypes of immune cells that may serve as potential therapeutic targets in BC. While this study is primarily descriptive in nature, the data compiled herein constitutes a valuable resource that enhances our understanding of the various cell types implicated in BC. It is our hope that these findings will contribute meaningful insights that can inform the development of future therapeutic strategies.

## Data Availability

Single cell data comes from the GSE161529 dataset of the GEO database, bulk RNA data comes from the GEPIA2 project. All public data are fully described in the methods section.

## Supplementary material

Supplementary Figure 1 The result of immunohistochemistry. **(a)** IHC of EC4 signature genes (CD74)in Normal and BC specimens. **(b)** IHC of EC5 signature genes (HSPG2,COL4A1,SPARC)in Normal and BC specimens.

Supplementary Figure 2 Characterization of EC subsets in BC subtypes. **(a)** UMAP embedding plot showing identified clusters of EC cells from Healthy,ER,HER2 and lymph-node metastases of ER of 16 patients. Cells were colored according to their cancer subtypes. **(b)** Kaplan-Meier plots showing the survival probability of BC patients with high and low expression levels of EC6 signature genes. Time (x-axis) is represented in months. (**c)** GSEA analysis showing the enrichment of DEGs in **Figures 1a–d**.

Supplementary Figure 3 Myeloid cell functional analysis. **(a)** Bar chart showing the relative proportion of each EC subset from different BC subtypes.Colors on the columns indicate cell type marked by legend. **(b)** Heatmap showing the BC subtypes distribution of each major endothelial cell type by Ro/e analysis.[Ro/e > 1]:+++,[0.8 < Ro/e <= 1]:++,[0.2 <= Ro/e <= 0.8]:+,[0 < Ro/e < 0.2]: +/-,[Ro/e = 0]:-. **(c)** Venn diagram showing overlapping and unique pseudotime genes of BC Myeloid subtypes from ER,HER and ER_LN. **(d)** The expression patterns of genes associated with the pseudotime. Colors on heatmap indicate the genes expression. Color from red to blue indicates a high to low expression. **(e–g)** Dot plot showing the functionally enriched pathways of unique pseudotime marker genes of each Myeloid subtype in ER,HER and ER_LN. Dot color and size indicate p value and gene number.

Supplementary Figure 4 Characterization of T/NK subsets and EC-based regulatory network for BC. **(a)** UMAP embedding plot showing identified clusters of T cells from Normal,ER,HER2 and ER_LN of 16 patients. Cells were colored according to their BC subtypes. **(b)** UMAP embedding plot showing identified clusters of T cells from Normal,ER,HER2 and ER_LN of 16 patients. Cells were colored according to their clusters. **(c)** Pseudotime analysis of selected T cell subsets for each BC subtypes. Distribution of the cells from different BC subtypes on each of the branches. (**d)** The expression patterns of genes associated with the pseudotime in T cell subsets. Colors on heatmap indicate the genes expression. Color from red to blue indicates a high to low expression. **(e)** Scatter plots of TF regulons based on specificity Z scores in CD8_Exh. **(f)** Heatmap showing the interaction intensity among major cell subsets from HER2. **(g)** Heatmap showing the interaction intensity among major cell subsets from ER_LN.
