# Supplementary figures and images for: Single-cell profiling identifies heterogeneity of the immune microenvironment in healthy, primary and lymph node metastatic BC
Source: Front Immunol. 2025 Nov 27;16:1690992. doi: 10.3389/fimmu.2025.1690992 (PMC12695843; doi:10.3389/fimmu.2025.1690992)

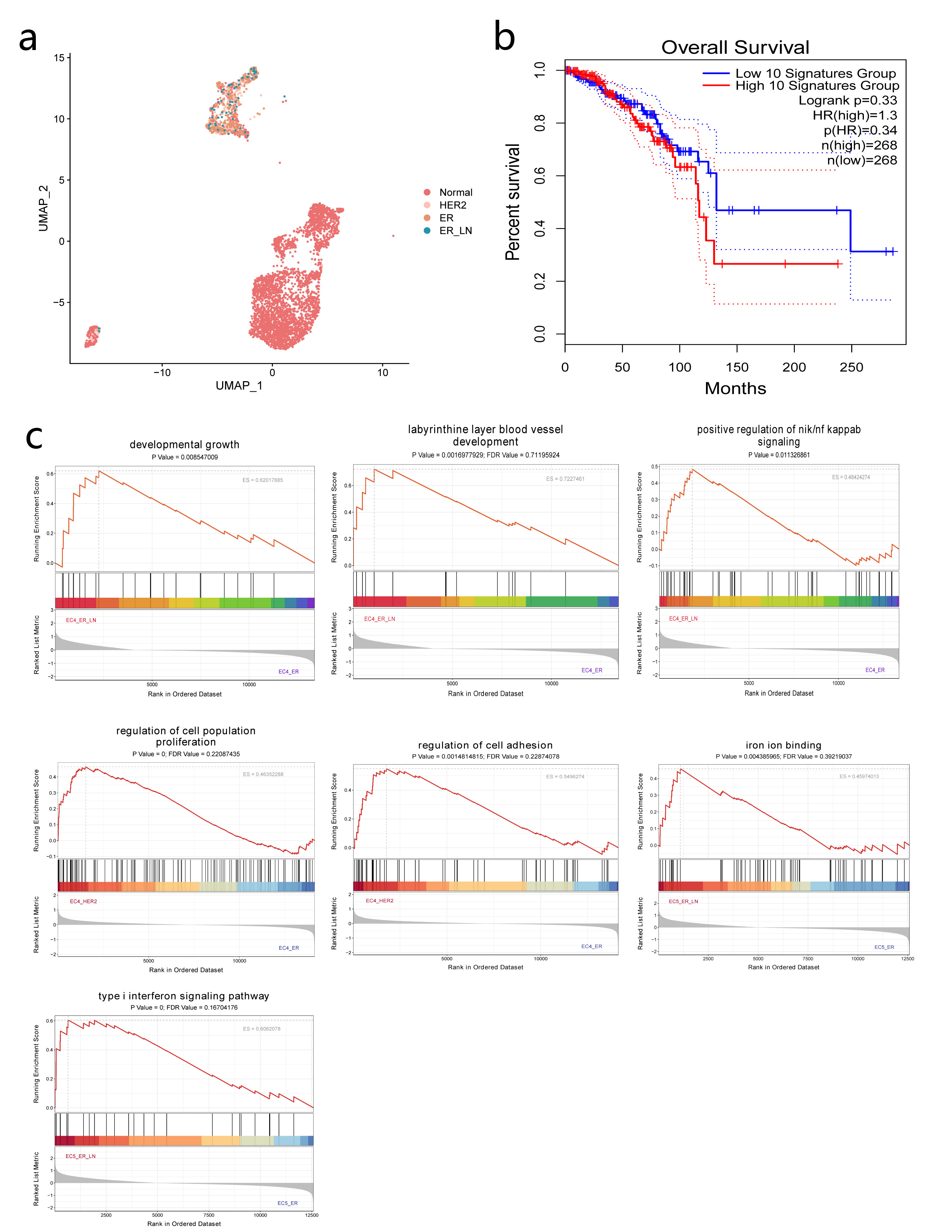

Supplement: Supplementary Figure 1 — The result of immunohistochemistry. (a) IHC of EC4 signature genes (CD74)in Normal and BC specimens. (b) IHC of EC5 signature genes (HSPG2,COL4A1,SPARC)in Normal and BC specimens. [file SupplementaryFile1.zip › Supplementary Fig.2.jpg]

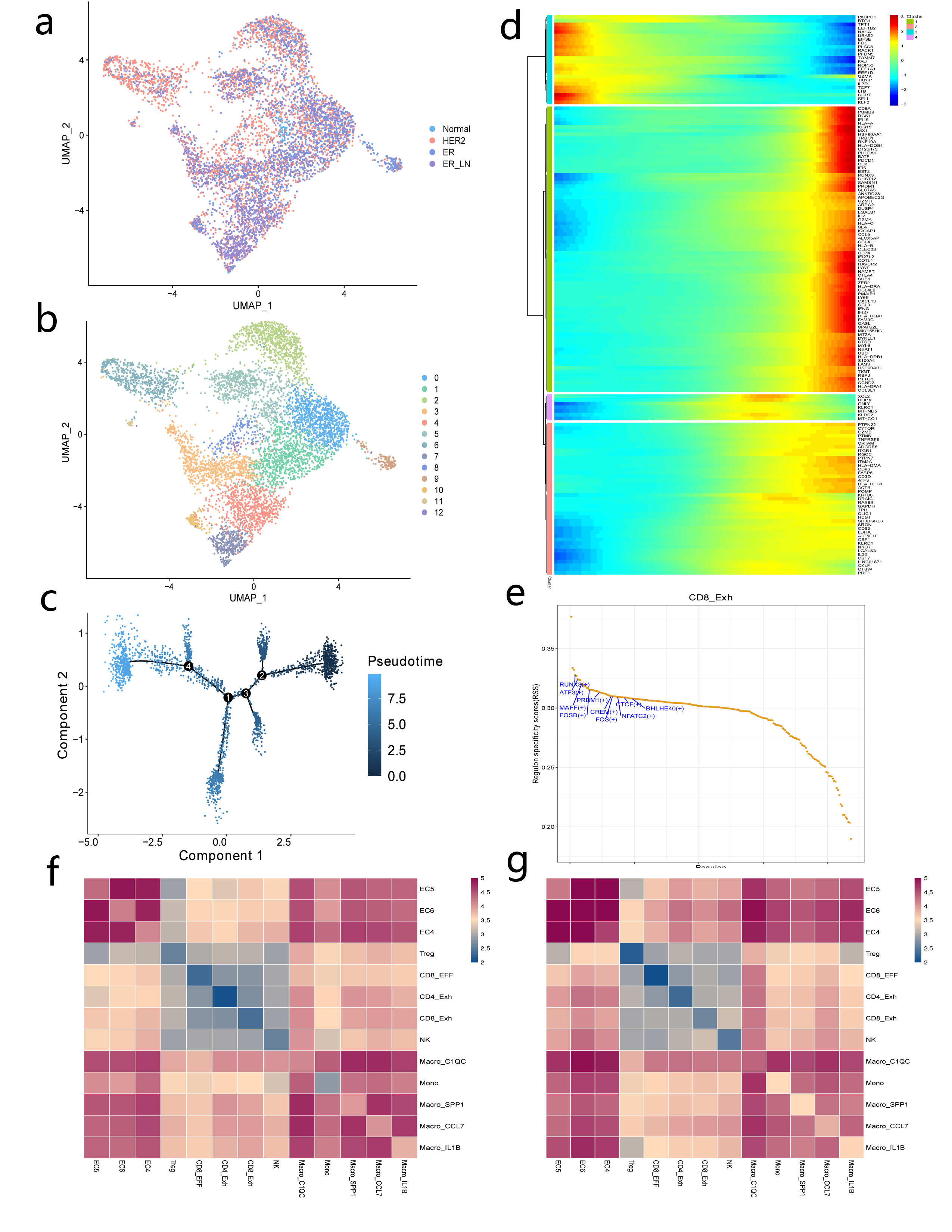

Supplement: Supplementary Figure 1 — The result of immunohistochemistry. (a) IHC of EC4 signature genes (CD74)in Normal and BC specimens. (b) IHC of EC5 signature genes (HSPG2,COL4A1,SPARC)in Normal and BC specimens. [file SupplementaryFile1.zip › Supplementary Fig.4.jpg]

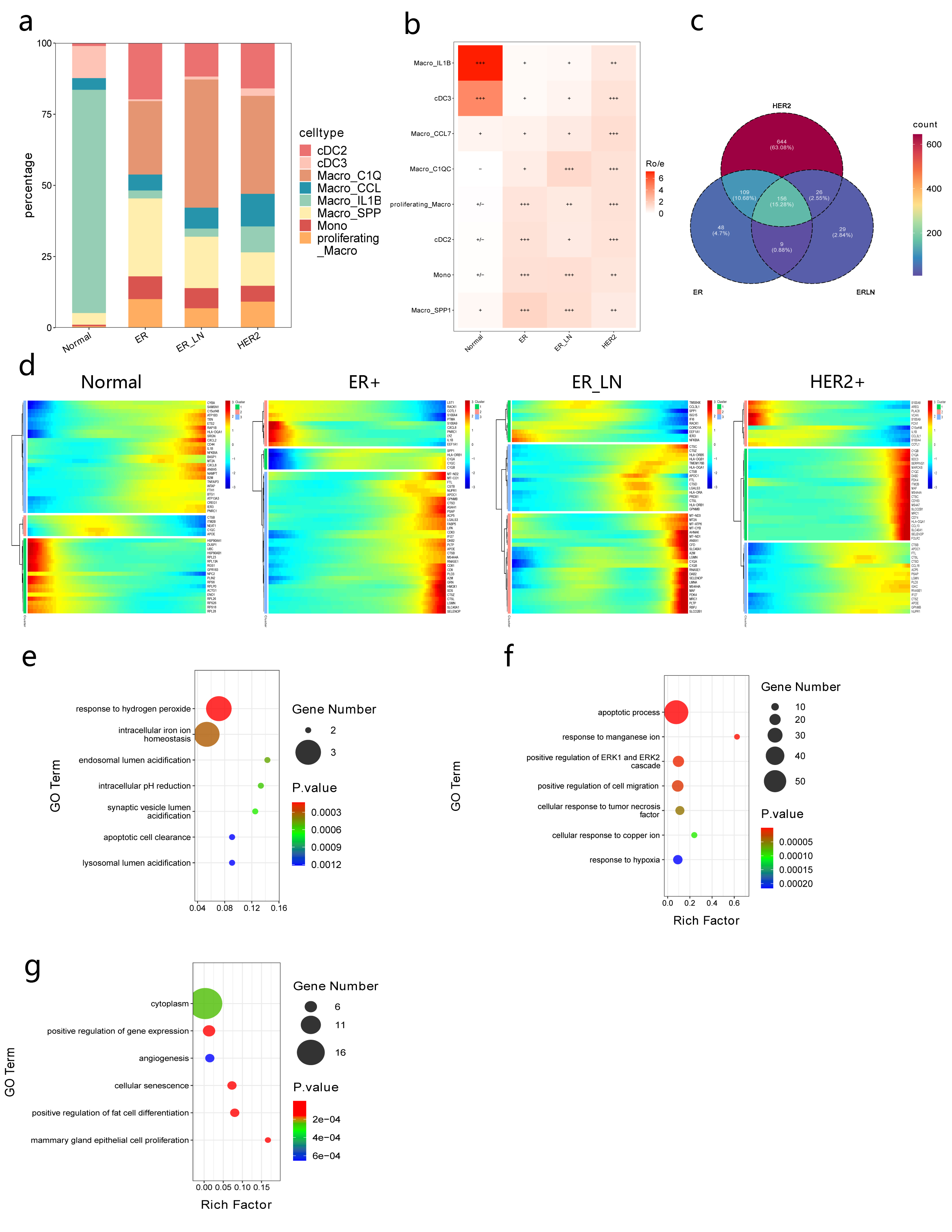

Supplement: Supplementary Figure 1 — The result of immunohistochemistry. (a) IHC of EC4 signature genes (CD74)in Normal and BC specimens. (b) IHC of EC5 signature genes (HSPG2,COL4A1,SPARC)in Normal and BC specimens. [file SupplementaryFile1.zip › supplementary Fig.3.jpg]

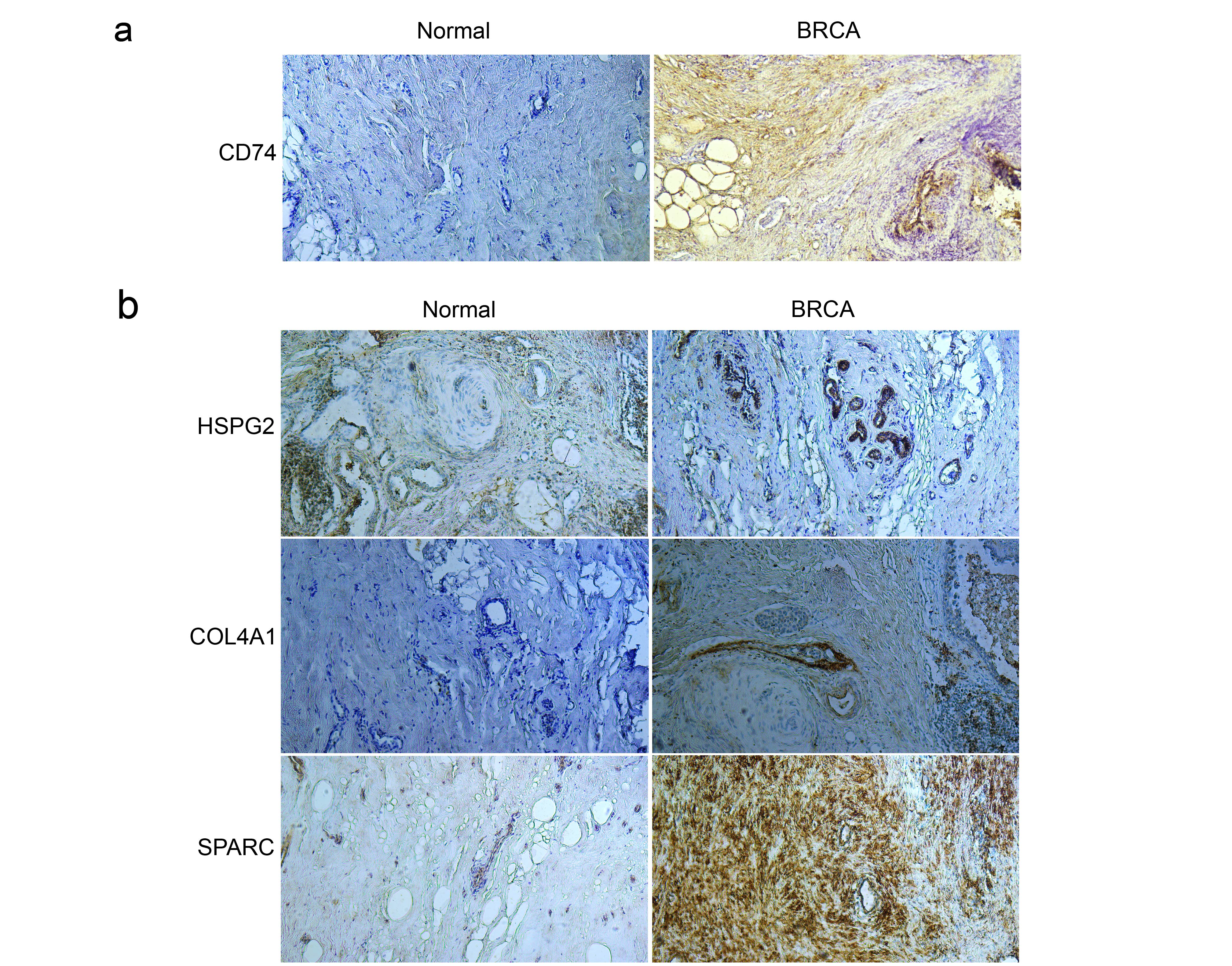

Supplement: Supplementary Figure 1 — The result of immunohistochemistry. (a) IHC of EC4 signature genes (CD74)in Normal and BC specimens. (b) IHC of EC5 signature genes (HSPG2,COL4A1,SPARC)in Normal and BC specimens. [file SupplementaryFile1.zip › Supplementary Fig.1.jpg]
